# Schistosome Feeding and Regurgitation

**DOI:** 10.1371/journal.ppat.1004246

**Published:** 2014-08-14

**Authors:** Patrick J. Skelly, Akram A. Da'dara, Xiao-Hong Li, William Castro-Borges, R. Alan Wilson

**Affiliations:** 1 Molecular Helminthology Laboratory, Department of Infectious Disease and Global Health, Cummings School of Veterinary Medicine, Tufts University, North Grafton, Massachusetts, United States of America; 2 Centre for Immunology and Infection, Department of Biology, University of York, York, United Kingdom; 3 Key Laboratory of Parasitology and Vector Biology, Ministry of Health, National Institute of Parasitic Diseases, Chinese Center for Disease Control and Prevention, Shanghai, People‘s Republic of China; 4 Laboratório de Enzimologia e Proteômica, Instituto de Ciências Exatas e Biológicas, Departamento de Ciências Biológicas, Universidade Federal de Ouro Preto, Ouro Preto, Minas Gerais, Brasil; International Centre for Genetic Engineering and Biotechnology, India

## Abstract

Schistosomes are parasitic flatworms that infect >200 million people worldwide, causing the chronic, debilitating disease schistosomiasis. Unusual among parasitic helminths, the long-lived adult worms, continuously bathed in blood, take up nutrients directly across the body surface and also by ingestion of blood into the gut. Recent proteomic analyses of the body surface revealed the presence of hydrolytic enzymes, solute, and ion transporters, thus emphasising its metabolic credentials. Furthermore, definition of the molecular mechanisms for the uptake of selected metabolites (glucose, certain amino acids, and water) establishes it as a vital site of nutrient acquisition. Nevertheless, the amount of blood ingested into the gut per day is considerable: for males ∼100 nl; for the more actively feeding females ∼900 nl, >4 times body volume. Ingested erythrocytes are lysed as they pass through the specialized esophagus, while leucocytes become tethered and disabled there. Proteomics and transcriptomics have revealed, in addition to gut proteases, an amino acid transporter in gut tissue and other hydrolases, ion, and lipid transporters in the lumen, implicating the gut as the site for acquisition of essential lipids and inorganic ions. The surface is the principal entry route for glucose, whereas the gut dominates amino acid acquisition, especially in females. Heme, a potentially toxic hemoglobin degradation product, accumulates in the gut and, since schistosomes lack an anus, must be expelled by the poorly understood process of regurgitation. Here we place the new observations on the proteome of body surface and gut, and the entry of different nutrient classes into schistosomes, into the context of older studies on worm composition and metabolism. We suggest that the balance between surface and gut in nutrition is determined by the constraints of solute diffusion imposed by differences in male and female worm morphology. Our conclusions have major implications for worm survival under immunological or pharmacological pressure.

Schistosomes are parasitic platyhelminths that cause the chronic, often debilitating disease called schistosomiasis, affecting several hundred million people around the world. Pairs of adult male and female worms reside either in the mesenteric veins of the intestine (*Schistosoma mansoni* and *S. japonicum*) or the veins enveloping the bladder (*S. hematobium)*. Worms are continuously bathed in blood and, unusual among parasitic helminths, they take up nutrients in two distinct ways — directly across the body surface and also by ingestion of blood into the gut. Much information on nutrient acquisition has been obtained from recent studies employing techniques such as RNA interference, proteomics, confocal microscopy, and laser capture microdissection. In this review, we assess the new observations against the background of older studies on worm composition and metabolism to evaluate the relative contributions of the schistosome body surface and gut in the uptake of different nutrient classes. These considerations have implications for worm survival under immunological or pharmacological pressure.

## Feeding Across the Body Surface

**Figure 1:**
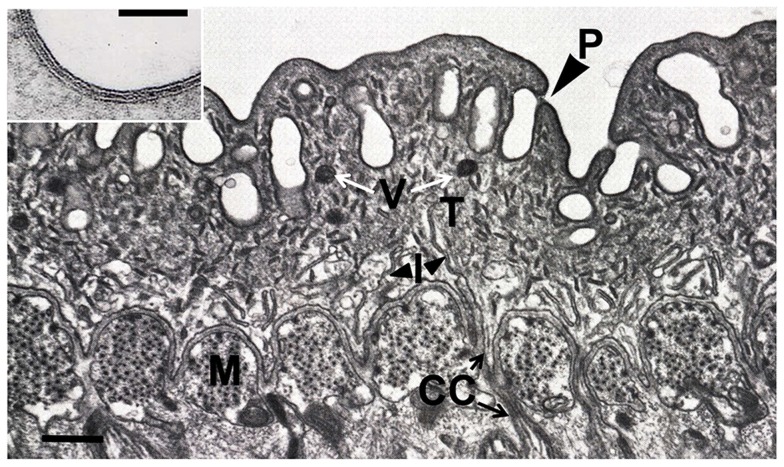
The tegument of adult *S. mansoni*. Electron micrograph of a transverse section through the male tegument (T) and underlying musculature (M). The outer half is pitted (P, large arrowhead) extending the surface area, but notably lacks the microvillus-like microtriches of tapeworms, which acquire nutrients only across the tegument. The basal plasma membrane shows numerous infoldings (I, small arrowheads) typical of transporting epithelia. The cell bodies that contain the biosynthetic machinery lie below the muscle layer and are joined to the syncytium by narrow microtubule-lined cytoplasmic connections (CC, arrows). The inset shows the tegument surface at higher magnification revealing the two closely apposed lipid bilayers comprising an inner plasma membrane and an outer membranocalyx. The latter originates as the secreted contents of multilaminate vesicles (V, white arrows) that are produced by the Golgi apparatus in the cell bodies. Scale bars = 1 µm.

Parasitic flatworms, both flukes and tapeworms, are covered by a syncytial layer of cytoplasm a few microns thick, which forms the interface between parasite and host (Figure 1). It is referred to as a tegument or neodermis (hence the taxon Neodermata) to distinguish it from the cuticle of the other major group of parasitic helminths, the nematodes. The tegumental syncytium originates by fusion of somatic cells around the developing larval (cercarial) embryo [1], [2]. Uniquely in schistosomes and other blood flukes, the outer surface of the tegument comprises two tightly apposed lipid bilayers [3] and nutrients must pass through both to reach the tegumental syncytium. Early experiments revealed that glucose entered the worm by this route [4], [5] while more recently the specific *Schistosoma mansoni* proteins mediating uptake have been characterized as schistosome glucose transporter proteins (SGTP) 1 and 4 [6]. Both are typical facilitated diffusion glucose transporters, exhibiting stereo-specificity, relaxed specificity for other hexoses, sodium independence, and substantial inhibition by cytochalasin B [6]. SGTP4 is localized uniquely to the apical tegumental bilayers [7] and brings glucose from the exterior into the tegumental cytoplasm. SGTP1 is detected in the tegumental basal membrane where it can transport glucose from the tegumental matrix further into the body of the worm [5]. Suppressing the expression of either SGTP1 or SGTP4, using RNA interference (RNAi), impairs the ability of the worms to take in glucose from their environment [8]. In addition, schistosomula whose glucose transporter genes have been suppressed fail to establish a robust infection in experimental animals [8].

Radiolabeled metabolite uptake experiments implied that the tegument was also virtually the exclusive route for amino acid import. These experiments involved worms that were suspended with their mouths excluded from medium containing either radiolabeled glycine or proline, versus worms fully immersed [9]. There was little difference in uptake between the two groups, suggesting that 80–100% of the absorption occurred via the tegument [9]. Under similar conditions, uptake of methionine was also shown to be primarily via the tegument [10]. However, the conclusion that the gut is not involved in amino acid uptake must be treated with extreme caution as worms in vitro appear very reluctant to open their mouths [11], so only tegument uptake will be measured by the above methods. Nevertheless, later work has demonstrated the existence of several tegumental amino acid transport systems in *S. mansoni* males, some highly specific for selected amino acids and others of broader specificity [12]. The molecular basis for one of these has been elucidated and designated schistosome permease 1 (SPRM1). SPRM1 is a cysteine disulphide-linked heterodimer consisting of a 72 kDa heavy chain protein (SPRM1hc) possessing a single transmembrane domain and a 55 kDa, multimembrane spanning, light chain (SPRM1lc) [13], [14]. The heavy chain is a chaperone that directs the light chain to the plasma membrane; here, SPRM1lc acts as the actual amino acid conduit. When expressed within *Xenopus* oocytes, SPRM1 facilitates the transport of all of the basic amino acids (viz., arginine, lysine, histidine) as well as leucine, phenylalanine, methionine, and glutamine [13]. Both SPRM1hc and SPRM1lc are widely distributed in schistosomes and their location in the apical tegumental membranes [13], [15] suggests that their role is to import amino acids from the bloodstream.

Like other parasitic platyhelminths, schistosomes are osmo-conformers, which means that water can leave or enter the worm's body according to external conditions. The aquaporins were discovered about 20 years ago to act as water conducting channels, and we now know that water movement across the schistosome tegument is mediated via SmAQP1, a ∼33 kDa aquaporin [16]. Indeed, quantitative proteomic analysis of adult tegumental membranes has revealed it as the single most abundant protein constituent in the surface [17], most likely in a tetrameric conformation like other aquaporins. The essential role of SmAQP1 was revealed when the gene was suppressed by RNAi; the worms were less viable in culture relative to controls while survivors were commonly stunted [16]. Another prominent feature of the exposed tegument surface is the presence of at least three phosphohydrolases, e.g., alkaline phosphatase (SmAP) [18]–[20]. These ectoenzymes cleave exogenous nucleotide phosphates [20], [21], thereby generating de-phosphorylated metabolites, which are more easily transported across membranes [22]. As schistosomes must salvage purines and nucleosides [23] since they cannot synthesize them de novo, the uptake of such nutrients through the tegument may be an important dietary source [22]. Recent proteomic analyses of isolated tegument fractions indicate that the tegument may be involved in the transport of additional metabolites. ATPase-associated Na/K, calcium and cation transporters, as well as anion and potassium voltage gated channels have been identified in tegument membrane preparations [24]. However, functional studies are required to confirm that the actual transport processes occur.

## Feeding via the Alimentary Tract

**Figure 2:**
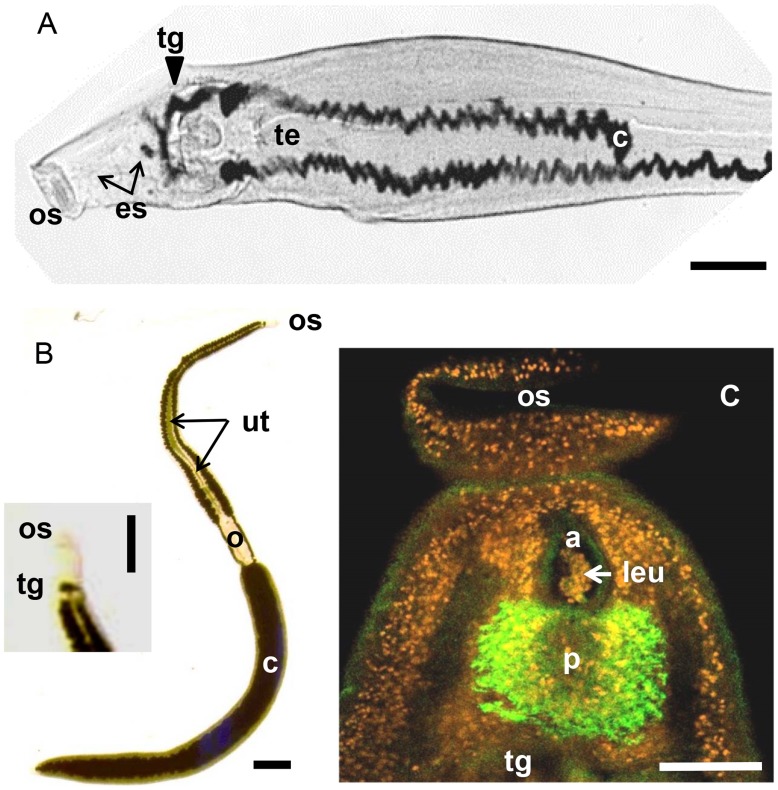
Layout of the alimentary tract. **A**. Male *S. haematobium* showing the distribution of black hemozoin pigment that delineates the lumen of the gut caecum (c). The mouth at the base of the oral sucker (os) opens onto a short esophagus (es, arrows) that empties into an initial transverse region of gut (tg, arrowhead). This bifurcates to pass round the testes (te) before reuniting approximately halfway down the body to continue to the extreme posterior where it ends blindly. **B**. Female *S. japonicum* from a rabbit with the gut lumen almost completely filled with dark hemozoin pigment. The layout is the same as for the male but with the bifurcated caeca (c) passing first around the egg-filled uterus (ut, arrows) and ovary (o), before uniting to form a single tube completely surrounded by vitelline follicles (inset, higher magnification, esophageal region). **C**. Confocal image of the anterior of a male *S. japonicum* from a rabbit host highlighting the esophageal gland (green), revealed by detection of esophageal-specific protein SjMEG-4.1, and the nuclei (false-colored orange) stained by DAPI. The short esophagus is lined with atypical tegument syncytium, the surface of the anterior compartment (a) being corrugated while that in the posterior (p), coincident with the gland, is extended ∼50-fold by thin plate-like extensions. Aggregates of host leucocytes (leu, arrow) are evident in the esophageal lumen. Scale bars: A, 0.75 mm mm; B, 0.5 mm (inset, 0.2 mm); C, 0.1 mm.

**Figure 3:**
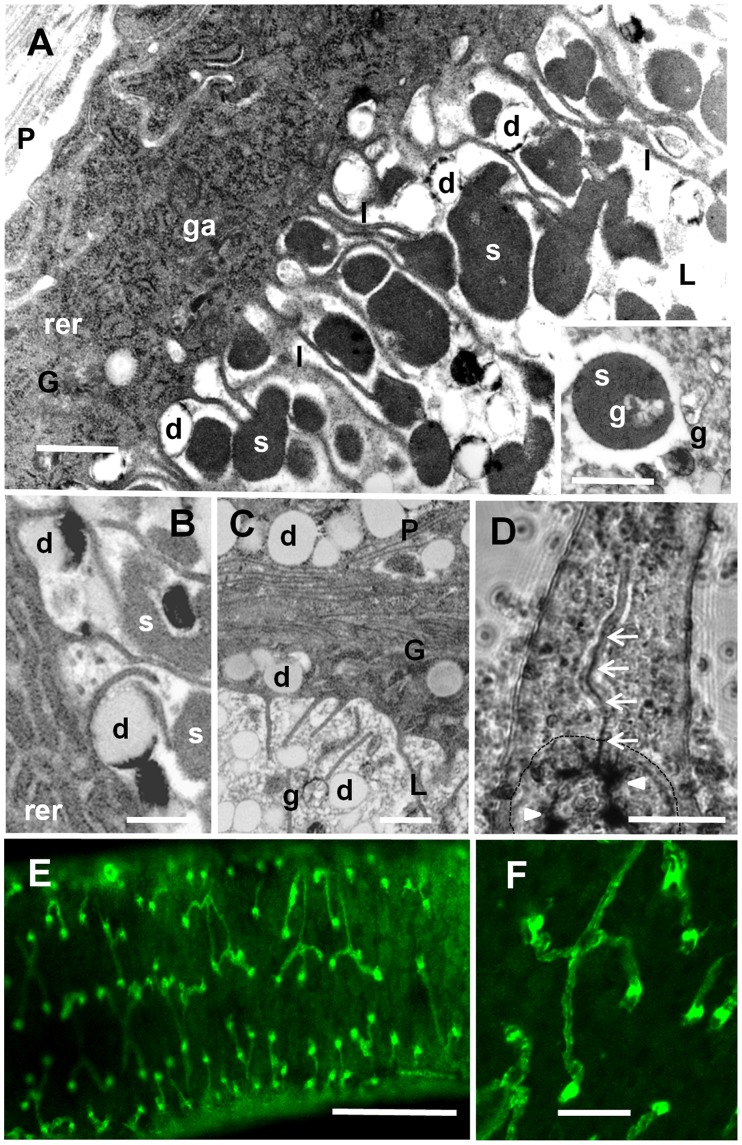
The gut and protonephridia. **A**. Transmission electron micrograph of the gut epithelium of an *S. mansoni* male. The cytoplasm of the syncytial gastrodermal epithelium (ga) is rich in rough endoplasmic reticulum (rer) and Golgi apparatus, typical of a cell synthesizing proteins and glycans for export; unlike the tegument it lacks obvious secretory inclusions. The luminal surface is extended by numerous thin lamellae (l) 3–5 microns long, in place of the conventional microvilli of an absorptive gut. Dense aggregates (“blobs”) of erythrocyte stroma (s) lie adjacent to the lamellae, together with paler lipid droplets (d). **Inset**, Stromal blob containing a hemozoin (Hz) pigment granule (g), with several more free granules adjacent. P, G and L denote Parenchyma, Gastrodermis and Lumen, respectively. **B**. Some (but not all) lipid droplets (d) have a dark ring of Hz around the periphery. **C**. The distribution of lipid droplets in the gut lumen, the epithelial syncytium and the surrounding parenchyma, is suggestive of transcytosis (i.e., the process of metabolite import into vesicles on one side of a cell followed by their release on the other side). **D**. Video frame from a feeding experiment during which an adult male *S. mansoni* regurgitated gut contents. Dark Hz demarcates the bifurcated gut (arrowheads) while a thin line of Hz (arrows) can be seen passing up the lumen of the posterior esophagus. (Numerous out-of-focus erythrocytes surround the worm. Dotted outline denotes ventral sucker.) **E**. Dorsal aspect of a female *S. japonicum* worm stained with FITC-labelled pea-nut agglutinin showing the bilateral distribution of flame cells (green dots) and protonephridial tubules running towards the main lateral collecting ducts on either side of the body. **F**. Higher magnification showing that each tubule terminates in a flame cell. The green dots are intense aggregates of O-glycan at the point where the flame cell connects to its tubule. Scale bars: A, 2 µm, inset 1 µm; B, C 0.4 µm; D, E 100 µm, F, 20 µm.

Given the demonstrable occurrence of solute movement across the tegument and the high levels of important metabolites in the blood, the relative contribution of the schistosome gut needs to be carefully evaluated. The alimentary tract comprises the mouth, a short esophagus lined with modified tegument [25], [26], and the absorptive gut, which runs to the farthest extremity of both males and females (Figure 2A, 2B); it is referred to as a caecum since it ends blindly. The posterior esophagus (stained in figure 2C) is surrounded by a gland that releases secretions into the lumen to interact with ingested blood, while the gut caecum is lined by an epithelial layer, the gastrodermis. This is syncytial, like the tegument, but with nuclei and biosynthetic machinery situated in the syncytial cytoplasm (Figure 3) [27]. The absence of an anus means that ingested blood and the residual products of digestion both pass through the same orifice. How these two processes of blood feeding and regurgitation of waste are coordinated is poorly understood.

### The dynamics of blood feeding

The first study to demonstrate unequivocally the participation of the gut in schistosome nutrition, involved the injection of ^3^H-leucine-labelled reticulocytes into infected mice [28]. Worms harvested by perfusion four days later were found to have incorporated the radiolabel, the females substantially more than males in keeping with their known greater appetites [28]. The leucine was extensively distributed in the tissues of the schistosomes, while the gut wall displayed radioactivity, suggesting amino acid uptake across the schistosome gastrodermis [28]. Later work with adults [29] and schistosomula [30] confirmed the finding. Note, however, that the autoradiographic technique used in these studies reveals the site(s) where labeled amino acid is incorporated into protein (i.e., biosynthesis), not the site of uptake, so the evidence is circumstantial.

Live *S. mansoni* worms viewed in the portal vasculature of an anaesthetized mouse were observed to face against the direction of blood flow, and feed in an energy efficient manner by simply opening and closing the oral cavity every 1–2 seconds [31]. Such feeding movements were either continuous for the observed period (60 minutes) or lasted ∼10 minutes to be repeated after a rest period of about the same duration [31]. Recent video recordings of *S. mansoni* males in vitro at 37°C revealed that erythrocytes were acquired from a dilute suspension by a “rapid grabbing motion” of the oral sucker, approximately four times per second [26]. A bolus of ingested blood accumulated in the lumen of the anterior esophagus. A wave of peristalsis then passed down the esophagus to drive the bolus to the posterior compartment and on into the anterior gut. The actual rate of blood consumption in sentient mice has been determined in a single elegant study involving intravenous administration of ^51^Cr-radiolabeled erythrocytes [32]. Male and female adult *S. mansoni* ingested some 39,000 and 330,000 erythrocytes per hour, equating to daily intakes of 105 nl and 880 nl of whole blood, respectively. Adjusted for packed cell volume, the female ingests ∼484 nl of plasma fluids per day, equivalent to 32.3 gut volumes, or 4.4 body volumes. The values for the male are 58 nl of plasma fluids equivalent to 7.7 gut volumes or 0.2 body volumes of fluid (Table S1). Time course measurements revealed that a steady state, where ingestion of label was balanced by its expulsion, was reached in approximately eight hours [32].

### The esophageal gland

Recent work on the functions of the esophageal gland has revealed that the esophagus has a central role in blood processing, not acting simply as a conduit [26]. Erythrocytes are rapidly lysed upon entry to the posterior esophageal compartment so that intact cells are seldom observed there [26], [27]. Remarkably, leucocytes are somehow tethered in the posterior lumen and can be observed, in varying states of degeneration, as a stationary plug (Figure 2C) [26]. This trapping could represent a mechanism to prevent, e.g., neutrophils from making a destructive oxidative burst in the gut lumen. Esophageal gland cell-bodies synthesize large numbers of crystalloid vesicles and release their contents into the lumen [26], [33]–[35]. By a combination of whole mount in situ hybridization and immuno-cytochemistry, a small number of esophageal gland-specific proteins has been identified. These include the Micro Exon Gene (MEG) products 4.1, 4.2 and 14 [26] and Venom Allergen Like (VAL) protein 7 [36]. They likely play roles in the cell tethering process and/or the extensive host cell lysis observed in the esophagus.

### Proteins

Digestion of lysed cell material and plasma constituents in the gut is largely extracellular. The pH of the gut has been measured at 5.0 or lower [37] and this may aid both in the unfolding of ingested proteins and in the activation of gut proteases (many of which function more efficiently in an acid environment) [38]. In this context, it has been suggested that lysosomes in the gastrodermal syncytium secrete their contents directly into the lumen to aid in the digestion of incoming blood components [11]. A battery of hydrolytic proteinases, all with an acidic pH optimum, has been identified in the regurgitated gut contents (vomitus) by proteomic analysis [11]. These include the cysteine endopeptidases, cathepsin L, cathepsin B, asparaginyl endopeptidase (legumain), and a cathepsin D-like aspartyl endopeptidase [39]; the localization of these proteases in the gastrodermis has been confirmed by immunocytochemistry [38], [40]–[43]. Exopeptidases, such as dipeptidylpeptidase (cathepsin C) [44] and proline carboxypeptidase homologs have also been identified [11]. A leucine aminopeptidease (LAP), with optimal activity at neutral pH, has been immunolocalized to the gastrodermal cells lining the lumen of adult schistosomes; LAP-immunoreactivity was considerably stronger within the gut of females versus males [45]. It has been proposed that this enzyme cleaves peptides that are taken in from the lumen into the cells. The resulting free amino acids are then distributed to other internal tissues [45].

The importance of these proteases for schistosomes has been demonstrated in some cases by suppressing the expression of their genes. For instance, cathepsin D-suppressed schistosomula appear unable to properly digest hemoglobin and do not survive to maturity when they are used to infect mice [43] and cathepsin B1-suppressed worms exhibit retarded growth compared to controls [46].

It has been proposed that the host specificity of blood-feeding organisms like schistosomes is determined by the molecular compatibility between the parasites' hemoglobin-digesting enzymes and the corresponding host species' hemoglobins [47]. In support of this, protease activities from *S. japonicum* (a schistosome with a very wide host range) cleave hemoglobin from bovine, sheep, and horse blood more efficiently than does the activity from extracts of *S. mansoni* (a schistosome with a limited host range, essentially confined to humans and some rodents) [48].

The quantitative data on blood ingestion [32] equate to a daily protein intake by adult females of ∼186 µg and males of only ∼18 µg (Table S1). Thus, a female worm ingests more than four times her dry weight in protein per day, whereas the male ingests only ∼0.2 times (Table S1). As the blood proteins are digested, the heme porphyrin ring within each hemoglobin subunit is released and detoxified by polymerisation into an inert, insoluble brown pigment hemozoin (Hz) [49], which gives the otherwise white schistosomes a dark appearance (Figure 2). The total heme intake per day is ∼0.56 µg for males and ∼4.8 µg for females (Table S1). However, the Hz content of a female is less than one-twelfth her daily intake of heme (at ∼0.375 µg heme per worm) while that of a male is about one-quarter (∼0.125 µg). This implies that there is little pooling of Hz pigment in the worm gut at steady state, the intake of hemoglobin being matched by Hz loss through regurgitation. The discovery of three iron-binding ferritin homologs in the vomitus indicates that some of the ingested iron may be sequestered for uptake into the gastrodermis (but see also the suggestion that a divalent metal transporter is localized in the tegument [50]). The identification of a calcium-binding calumenin homolog in vomitus suggests that the gut also plays a role in the regulation of calcium ions [11].

### Lipids

Schistosomes cannot synthesize fatty acids or sterols de novo, so must obtain them in the diet and subsequently modify them to meet their metabolic need. The total lipid content of adult schistosomes is just over one-quarter of dry weight, but due to the differences in body mass and size of blood meal, the male ingests only about 2.5% of his lipid content per day, whilst the female ingests 50% (Table S1). For males at least, this implies significant lipid storage capacity, potentially in the parenchyma cells where lipid droplets are very evident. The female ingests more lipid in total than the male but since lipid is the same proportion of dry weight in both sexes, she must metabolize (or expel) more lipid on a daily basis than the male.

Low density lipoprotein (LDL) has been reported to bind to the tegument of *S. mansoni* schistosomula [51], [52] and LDL-binding proteins of varying molecular weights have been identified in different schistosome extracts [53], [54]. However, endocytosis of surface labeled lipid has never been observed. In addition, no clear LDL-receptor homolog has been identified in any analysis of the schistosome tegumental proteome [15], [18], [19], providing no evidence that lipid import occurs across the tegument. Regarding the gut, proteomic analysis of vomitus identified a Niemann Pick type C2 protein (NPC2) homolog [11]. NPC2 helps to traffic cholesterol and other sterols and glycolipids in eukaryotic cells; its presence in the schistosome gut suggests a similar function. Several saposin homologs were also found, and one of them, designated Sm-SLP-1, has been localized to the adult gastrodermis by immunocytochemistry [55]. By analogy with saposin function in other systems, these proteins may act to sequester lipids in the parasite gut lumen for uptake by gastrodermal cells [11]. These findings suggest that the gastrodermis plays a prominent role in lipid and fatty acid uptake.

### Carbohydrates

It is clear that glycogen is a major storage polysaccharide accounting for 10.5% of the dry weight of males but only 2.8% of females [56], perhaps reflecting the male's greater muscular effort when transporting the female. However, the amounts of glucose ingested in blood per day are small, 0.11 µg for the male and 0.88 µg for the female (Table S1). This has to be set against the well-documented consumption of glucose by worms in culture and the concomitant secretion of similar amounts of lactate. For the male, the figure is 426 µg per day and for the female 115 µg, (representing 4.5 and 2.9 times the respective dry weights) [57]. This amounts to a massive 3872-fold greater consumption than ingestion by the male and 137-fold by the female, emphatically confirming the tegument as the site of glucose uptake, with the gut making a negligible contribution overall. Nevertheless, the identification of a glycan 1,4 beta glucosidase homolog in vomitus implies that complex carbohydrates can be digested in the gut [11]. Furthermore, incubating adult worms in vitro with ^3^H-glucosamine for 30 minutes revealed rapid accumulation of label in gastrodermal cells, suggesting the gut might also be a prominent site for uptake of amino sugars [29], but again with the caveat about autoradiography as a detection method.

### Gastrodermal function

The real problem with the investigation of gastrodermal function is the inaccessibility of the tissue; no one has yet reported isolation of intact gastrodermis from the solid schistosome body. However, in a novel approach, slices of gastrodermal tissue from adult female *S. mansoni* and *S. japonicum* were isolated using laser microdissection microscopy to obtain gene expression profiles [58], [59]. As might be anticipated, transcripts encoding a range of endo- and exo-peptidases were abundant in the gastrodermis and expression of a number of saposins together with the NPC2 homolog was evident. A single amino acid transporter homolog was detected but no glucose transporter homologs [58], [59]. Genes associated with membrane trafficking through endocytic compartments and lysosomes were also expressed, reflecting the absorptive nature of the gastrodermis. In this context, it is notable that a recent feeding study with fluorescent-labeled dextran (MW 10 kDa) co-administered with an erythrocyte suspension to 28-day-old worms in vitro [11], showed that by one hour the dextran had reached the furthest extremity of the gut. By 24 hours, it had entered the gastrodermis, where it accumulated in ∼2 µm diameter aggregates. This is the first evidence for macropinocytosis, or receptor-mediated endocytosis, at the luminal surface of the gut epithelium [11]. The genes encoding the molecular machinery of endocytosis (e.g., clathrin heavy and light chains, assembly proteins AP2 and AP180) are present in the schistosome genome [60]; are they expressed in the gastrodermis?

## Waste Disposal

A review of feeding would be incomplete without a consideration of how the residues from ingested blood and the waste products of metabolism are expelled. The lack of an anus means that the mouth must also serve as a portal for egress while the involvement of the protonephridial system ramifying through internal tissues has largely been neglected.

### Regurgitation

Following hemoglobin digestion in the schistosome gut, peptides and heme are released [49]. Heme is a complex of iron with protoporphyrin IX and an essential molecule for most organisms [61]. Evidence that heme is used in schistosome metabolism comes from parasites fed reticulocytes in which the heme moiety of hemoglobin was radiolabeled; they later display some radioactivity (∼20%) in their protein fraction [30]. However, due to its potentially pro-oxidant effects, the excess heme that may cause cell damage is converted to hemozoin (Hz) [61], envisioned as a lattice of hydrogen-bonded heme dimers, linked in a head-to-tail manner [62]. Hz formation has been proposed to occur at the surface of lipid droplets in the schistosome gut lumen [49]. However, Hz particles can also be seen within hemoglobin aggregates undergoing digestion (Figure 3A). As Hz is lipophilic, its presence in lipid droplets may simply reflect its natural partitioning there. In this situation, we suggest that as the luminal lipid droplets (some ringed with Hz) (Figure 3A, 3B) continue to be degraded by gut enzymes, eventually all that is left is an insoluble Hz core, adding to the dark, inert Hz accumulations in the schistosome gut (Figure 2).

The description of Hz expulsion from adult worms as “regurgitation” and naming the released material as “vomitus” suggests a violent process. However, worms in vitro are rarely seen voiding Hz unless they are subjected to a chemical, osmotic, or temperature shock [11], [63], [64]. Indeed, the one direct series of observations on adult worms in the intestinal vasculature of mice [31] makes no mention of Hz regurgitation. A simple explanation is that the approximately 26-fold reduction in overall mass when heme polymerises to Hz upon proteolysis of hemoglobin (Table S1) means there is much less material to be regurgitated than was ingested, so it will occur infrequently. In the recent video analysis of male *S. mansoni* feeding in vitro [26], regurgitation was recorded but did not involve reverse peristalsis of the esophagus. Instead, intense motor activity in the anterior gut, followed by relaxation of the esophageal wall muscles, resulted in the movement of a thin dark line of vomitus up the esophageal lumen and out through the mouth [26] (Figure 3D). It is unclear whether Hz can be selectively eliminated during regurgitation, while important cathepsins, peptidases, saposins, and a variety of carrier protein homologs are retained [11], [63], [64]. However, the Hz expelled into the bloodstream of infected animals [65], [66] has been proposed to exert an immunomodulatory effect on the host [67].

Besides Hz, other molecules are released by schistosomes and can be detected in the circulation of infected individuals [68], [69]. Most prominent among them are two O-linked glycoproteins called circulating anodic antigen (CAA) and circulating cathodic antigen (CCA) [70], which form a mucin-like coat over the gastrodermis. The major O-linked chains of CAA consist of long, negatively charged repeats of 1–6-linked N-acetylgalactosamine residues substituted with 1–3-linked glucuronic acid [71]. The glycan moiety of CCA consists of linear repeats of the Lewis x (Le^x^) trisaccharide that comprise galactose β1–4 linked to N-acetlyglucosamine, the latter substituted with α1–3 linked fucose [72]. The polypeptide backbone of CCA is reputed to be a ∼39 kDa protein [73] but that of CAA is not known. Detection of CAA in serum and CCA in urine has been employed for immunodiagnosis of schistosomiasis [74]–[76]. The release of these glycan structures into the host bloodstream may have immunoregulatory consequences. Since carbohydrates with repeating Lewis x units are also found on circulating neutrophils of the host, the antigenic poly-Lewis x polysaccharide of CCA may induce auto-antibodies against granulocytes, causing the mild to moderate neutropenia observed during schistosome infection [72]. CAA has been shown to interact in vitro with the collagen-like domain of the first complement component Clq [77]. In this way it could interfere with complement cascade activation and/or the binding of Clq to its receptor.

### The protonephridial system

The protonephridial system in schistosomes, ramifying throughout the parenchyma of both sexes, is a bifurcated highly branched network of tubules (terminal diameter ∼2 um), each branch ending in a flagellated flame cell (∼10 µm by 3 µm) (Figure 3E, 3F). The beating of the ∼50 flagella inside the barrel, composed of interdigitations between the cap cell and first tube cell, is believed to provide the motive force to suck interstitial fluids through the desmosome-like filter into the tubule lumen [78]. Cilia are found in some wider collecting tubules, presumably to aid fluid passage towards the posterior terminal pore [79]. The minute size of these composite elements and the small volume of the system overall means there is a dearth of information about the physiology of the system in any flatworm. From the localization of three proteins to the adult worm tubules, as part of other studies (Sm bone morphogenic protein, ER-60 cysteine protease and protein disulphide isomerase, [80]–[82]) we can infer the presence of signaling systems, and the synthesis of proteins for export. The fluorescent marker resoruffin, which diffuses into the worm across the body surface, is subsequently excreted via the protonephridial tubules [83], providing direct evidence for the involvement of this system in the excretion of metabolic wastes and xenobiotics. Furthermore, this process is likely facilitated by a multi-drug resistance protein [83], two copies of which are present in the *S. mansoni* genome [84].

## Conclusion

**Figure 4:**
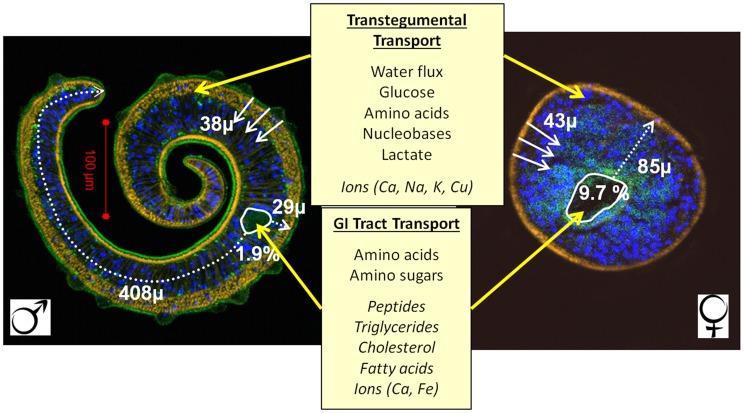
Pathways for diffusion of nutrients in male and female schistosomes. Confocal images of stained adult male (left) and female (right) schistosome cross sections at the same magnification (yellow, phalloidin for actin; blue, DAPI for nuclei; green, tegument and gut). The parameter values are the mean of 12 females and 11 males in near perfect cross section. Areas and distances were measured using the polygon line and path tools in the Analyzing Digital Images package from the Lawrence Hall of Science, Berkeley, California (http://www.globalsystemsscience.org/software/download). Solid white arrows show diffusion distances from the tegument surface to the midpoint, dotted white arrows those from the gut lumen to the furthest extremity. The gut lumen of the female occupies 9.7% of the cross section, that of the male only 1.9%. Nutrients diffusing from the male gut would have to travel >400 µm to reach the furthest tissues whereas the maximum distance from the female gut is only 85 µm. The distance a nutrient has to diffuse from the tegument surface to the tissue midpoint in both male and female is very similar at ∼40 µm. Central boxes list metabolite classes that are transported across the tegument (upper box, yellow arrows) or via the gastrointestinal tract (lower box, yellow arrows). Metabolites that have been shown experimentally to be transported are listed in the upper group in each box. Those that have been inferred to be transported from proteomic, transcriptomic, or other work are indicated by italics.

Due to their importance as agents of disease morbidity, schistosomes are the most widely researched parasitic flatworms and quite possibly helminths in general. The habitat of adult worms in the bloodstream provides a perpetual and rich source of nutrients. Earlier research on parasite composition emphasised the sheer scale of the feeding process, especially in the female worm. More recent studies have highlighted the mechanisms which mediate this process; a synopsis both of current knowledge and areas of uncertainty is provided in Box 1 and Figure 4. Our contention is that the success of schistosomes as pathogens can be attributed in part to the unique and complementary balance between active feeding via the alimentary tract and nutrient uptake across the body surface.

**Box 1. A Synopsis**

**Body shape:** The disparity between the dorso-ventrally flattened male and the almost cylindrical female (Figure 4) undoubtedly has a marked influence on their respective metabolic predispositions. The physical constraints imposed on the diffusion of small molecules through tissues must favour the tegument as the predominant site of nutrient uptake in males, whereas an even balance between gut and tegument is more likely in females.

**The tegument and gut epithelia:** Neither the pitted, sponge-like tegument nor the gastrodermis with its surface lamellae are typical of the solute-absorbing, microvillus-bearing surfaces found in the tissues of higher animals. This must reflect the ancient evolutionary origins of the schistosomes, with a triploblastic but acoelomate body plan, so novel aspects of nutrient uptake might be anticipated. Secretion of lysosomal enzymes into a gut lumen that operates at <pH 5.0 is one example.

**The feeding cycle:** Another male/female disparity is the size of the oral/esophageal pump relative to the blood consumed. The pump in the female is only one-fifth the size of that in the male but must move 8.5 times the volume of blood, so needs to work 40 times as hard. This makes it highly likely that females feed continuously, with short interruptions for regurgitation of hemozoin, whereas males feed intermittently (the male cannot ingest blood while using its oral sucker to transport a female along blood vessels). A feeding/digestion/regurgitation cycle in males would explain why they come in various shades of brown while females are more uniformly black.

**Esophageal gland function:** The demonstration that the esophageal gland plays a central role in blood processing has added an extra dimension to schistosome nutrition. A small number of products has already been identified, but characterisation of the secretions and definition of the roles of individual proteins presents a major challenge. How erythrocytes are lysed, leucocytes are trapped and damaged, yet blood coagulation does not block the esophagus are the principal questions to be tackled.

**Water-salt balance:** Worm bodies do not become bloated as they feed, so the ingested plasma fluids (water and salts) must be continuously expelled—in the female, the equivalent of 4.4 body volumes per day. Since vomitus is a thin and infrequent trickle, regurgitation can only play a minimal role. That leaves the protonephridial system, which ramifies through internal tissues but is poorly understood, or the tegument, where aquaporin is very abundant in the surface membranes. As lactate is expelled via aquaporin pores [65], excess water may travel the same route, and the Na/K pump in the tegument membranes is also likely involved. The 8-fold disparity in plasma fluid intake means that the female has a much greater task than the male, and this must represent a significant metabolic burden.

**Amino acids and proteins:** No quantitative estimates of amino acid uptake by medium depletion are available to compare with those for glucose. However, amino acid transporters are present in the tegument surface and may be especially important in male tissues distant from the gut (Figure 4). Nevertheless, in the female the amount of blood protein ingested (4.5 times dry weight), coupled with shorter diffusion distances, likely make it the dominant source. Undoubtedly this reflects the much greater exported biomass of eggs from females (∼300 per day in *S. mansoni*, ∼2000 in *S. japonicum*) compared to that of sperm produced by males. The current paucity of information on the gastrodermal transcriptome and proteome is an obstacle to understanding gut function. The recent paper on schistosome organ isolation may provide a way forward [66].

**Carbohydrates and energy metabolism:** The tegument is clearly the predominant route of glucose uptake for ATP generation in both sexes. A minor transport component is possible via the gut, involving special sugars (e.g., for glycan synthesis). It is notable that females take up less glucose but ingest greater amounts of amino acids (as protein) than males. The possibility that the amino acid carbon skeleton is used in females for ATP generation has not been explored, although the requisite transaminases and aminotransferases are encoded in the genome.

**Lipids:** Despite some effort, there is no significant evidence for uptake of essential lipids across the tegument, so we must assume the gut is the dominant route in both sexes; indeed, the presence of the membranocalyx may preclude lipid entry to the tegument. The multiplicity of saposins in the gut lumen, plus the Niemann-Pick type C (NPC) cholesterol transporter homolog reinforces this conclusion. Lipid droplet transcytosis across gut epithelium is a possibility, but has not yet been demonstrated by feeding experiments, as performed with dextran.

**Epilogue:** Once established in the bloodstream, schistosomes are hard to eliminate. The tegument and alimentary tract are the principal interfaces with the host so a better understanding of the functions they perform should provide avenues for more effective interventions. Is there scope for new diagnostic markers among the gut secretions expelled in vomitus? Are the transport proteins in both tegument and gut membranes suitable targets for pharmaceutical intervention? Do the esophageal gland secretions represent a novel cohort of vaccine candidates that can be targeted to disrupt blood feeding? Are females, with their greater nutritional requirements, more susceptible targets than males, a feature that would reduce transmission?

## Supporting Information

Table S1 **Dynamics of blood feeding in intravascular schistosomes.**
(XLSX)
